# Myocardial Performance Improvement After Iron Replacement in Heart Failure Patients: The IRON-PATH II Echo-Substudy

**DOI:** 10.3390/jcm14124048

**Published:** 2025-06-07

**Authors:** Raúl Ramos-Polo, Maria del Mar Ras-Jiménez, María del Carmen Basalo Carbajales, Sílvia Jovells-Vaqué, José Manuel Garcia-Pinilla, Marta Cobo-Marcos, Javier de Juan-Bagudá, Cândida Fonseca, Josep Francesch Manzano, Andreea Eunice Cosa, Sergi Yun-Viladomat, Cristina Enjuanes, Marta Tajes Orduña, Josep Comin-Colet

**Affiliations:** 1 Cardiology Department, Heart Institute, Bellvitge University Hospital, L’Hospitalet de Llobregat, 08907 Barcelona, Spain; rramosp@bellvitgehospital.cat (R.R.-P.);; 2 Bio-Heart Cardiovascular Diseases Research Group, Bellvitge Biomedical Research Institute (IDIBELL), L’Hospitalet de Llobregat, 08908 Barcelona, Spain; sjovells@idibell.cat (S.J.-V.); jfrancesch@idibell.cat (J.F.M.); aeunice@idibell.cat (A.E.C.); syunvi@gmail.com (S.Y.-V.); 3 Community Heart Failure Program, Cardiology Department, Bellvitge University Hospital, L’Hospitalet de Llobregat, 08907 Barcelona, Spain; mar.ras.jimenez@gmail.com; 4 Centro de Investigación Biomédica en Red de Enfermedades Cardiovasculares (CIBERCV), Instituto Salud Carlos III, 28029 Madrid, Spain; marlucale41@gmail.com (J.M.G.-P.); martacobomarcos@hotmail.com (M.C.-M.); javierdejuan166@hotmail.com (J.d.J.-B.); 5 Department of Clinical Sciences, School of Medicine, Universitat de Barcelona, 08007 Barcelona, Spain; 6 Department of Internal Medicine, Bellvitge University Hospital, L’Hospitalet de Llobregat, 08907 Barcelona, Spain; 7 Cardiology Department, Virgen de la Victoria Hospital, 29010 Malaga, Spain; 8 IBIMA-Platafrma BIONAND, 29590 Malaga, Spain; 9 Department of Medicine and Dermatology, Universidad of Málaga, 29071 Malaga, Spain; 10 Cardiology Department, Puerta de Hierro University Hospital, 28222 Majadahonda, Spain; 11 Cardiology Department, Instituto de Investigación Sanitaria Hospital 12 de Octubre (imas12), 12 de Octubre University Hospital, 28041 Madrid, Spain; 12 Department of Medicine, Faculty of Biomedical and Health Sciences, Universidad Europea de Madrid, 28670 Madrid, Spain; 13 Heart Failure Clínic, S. Francisco Xavier Hospital, Department of Medicine, ULS Lisboa Ocidental, NOVA Medical School, Universidade Nova de Lisboa, 1150-190 Lisboa, Portugal; mcandidafonseca@gmail.com

**Keywords:** chronic heart failure, comorbidities, iron deficiency, echocardiography, myocardial work

## Abstract

**Background:** Iron deficiency (ID) is a commonly seen comorbidity in heart failure (HF) patients. It is often associated with a poor prognosis and impaired physical capacity. The functional limitations linked to ID may lead to cardiac function abnormalities. The functional limitations linked to ID may lead to cardiac function abnormalities, that can be reversible after iron repletion. Some echocardiographic parameters, such as global longitudinal strain (GLS), myocardial work (MW) and its derivatives constructive work (CW), wasted work (WW) and work efficiency (WE), may be of added value in advanced cardiac performance assessment. **Methods**: IRON-PATH II was a multicenter, prospective and observational study designed to describe the pathophysiological pathways associated with ID. The echo-substudy included 100 HF patients that had undergone a specific pilot echocardiographic evaluation. Patients had a left ventricular ejection fraction (LVEF) ≤50%, were in stable clinical condition and on standard HF medication with hemoglobin ≥11 g/dL. The final cohort included 98 patients. **Results**: The ID group showed worse cardiac function, with lower GLS (−8.5 ± 9% vs. −10 ± 10%), WE (74 ± 10% vs. 80 ± 10%) and MW (665 [453–1013] vs. 947 [542–1199] mmHg%), as well as higher WW (290 [228–384] vs. 212 [138–305] mmHg%) and lower RV free wall strain (−13 [−20–(−11)]% vs. −17 [−23–(−14)]%). Following iron repletion, ID patients demonstrated improved LV (GLS, MW, WE and WW) and RV performance (RV free wall strain), aligning with non-ID patients (all *p*-values >0.05 compared to the non-ID group). **Conclusions**: Among HF patients with reduced LVEF, ID was associated with worse myocardial performance in both the LV and RV. All the alterations seen were reversible after intravenous iron repletion.

## 1. Introduction

Iron deficiency (ID) is highly prevalent among heart failure (HF) patients [1]. Regardless of the presence of anemia, ID reduces the energy efficiency of non-hematopoietic tissues with high energy demands [2]. Thus, ID affects the function of the cardiomyocytes by impairing the mitochondrial respiration chain, ATP production and contractility [3]. The decline in myocardial function due to ID has a pathophysiological basis, which has been explored in both human and animal models [4]. Therefore, anemia and ID directly affect cardiac function since they lead to a decrease in preload and the size of the left ventricular cavity [5].

Iron repletion in patients with HF and a left ventricular ejection fraction (LVEF) of less than 50% is associated with improvements in functional capacity and the quality of life and a reduction in rehospitalization [6,7,8,9,10]. However, the mechanisms by which iron replenishment improves exercise capacity and the quality of life remain unclear.

Although previous studies have shown that iron therapy may be linked to enhancements in cardiac function [5,11], the surrogate parameters of myocardial function used in these studies have several limitations. The first one, LVEF, is a volume-derived index that relies on geometric assumptions and is load-dependent [12]. Second, the conditions of elevated pre- or after-load directly affect GLS despite speckle-tracking-derived longitudinal strain offering lower inter-and intra-observer variability [13].

Regarding the limitations, the development of new echocardiography software has provided access to non-invasive assessments of highly valuable and more accurate information on cardiac physiology via the myocardial work (MW) index directly obtained by measuring the area of the pressure–strain loop. This is also the case for its derivatives constructive work (CW) and wasted work (WW) [14]. Finally, the non-invasive assessment of ventricular–arterial coupling (VAC) may be useful for a more complete cardiovascular performance profiling in this setting.

Interestingly, none of the previous studies that have attempted to assess the impact of iron status on cardiac function parameters have explored the impact of iron deficiency on myocardial performance parameters. This information is crucial to explaining the mechanisms that produce the worse exercise capacity observed in patients with ID [15]. Finally, the reversibility of the potential myocardial performance alterations associated with ID after iron replacement is undetermined.

Given the knowledge gap in this field and the limitations of previous studies, the IRON-PATH II echo-substudy was designed to define cardiac remodeling and myocardial deformation associated with systemic and tissue ID in patients with HF and reduced LVEF when compared to patients with HF without ID. Secondly, we aim to explore changes in myocardial function after iron replacement by using echocardiography.

## 2. Materials and Methods

**Study design.** IRON-PATH II (New pathophysiological pathways involved in iron metabolism disorder in hear failure: The IRON PATH II investigator-initiated Study, NCT05000853) was a multicenter, prospective, observational and investigator-initiated study aimed at characterizing the biological pathways involved in ID in HF patients and its association with laboratory biomarkers and clinical outcomes. The IRON-PATH II echo-substudy was designed to describe the echocardiographic features of HF patients according to ID and the effects of intravenous ferric carboxymaltose repletion on echocardiographic parameters.

The IRON PATH II study included a total of 210 HF patients (80 patients without ID and 130 patients with ID) recruited in 7 centers across Spain and Portugal between August 2021 and May 2023 and followed for a fixed period of 12 months. The echo-substudy included a subset of 100 patients on whom a specific echocardiographic evaluation was performed. All of them were recruited in the coordinating center.

The IRON-PATH II study was sponsored by CSL Vifor with coordination by Bellvitge Biomedical Research Institute (IDIBELL). The sponsor did not participate in the design of this study nor data analysis. An independent data and safety monitoring committee reviewed the data every 6 months. This study was approved by the local ethics committees for clinical research at each participating center and was conducted in accordance with the principles of the Declaration of Helsinki. All the patients gave written informed consent before entering this study.

The echo-substudy was included as a specific analysis within the IRON-PATH II project for the patients included at Bellvitge University Hospital. Safety was reported by local investigators in accordance with the current legislation that regulates pharmacovigilance in Spain and Portugal. Endpoint adjudication for the primary outcome components of this study was conducted on an ongoing basis by an independent endpoint committee blinded to the allocation of patient groups according to reports of serious events (death, readmission) by local investigators.

**Patients.** Patients with HF and an LVEF ≤50% according to the European Society of Cardiology (ESC) diagnostic criteria [16], receiving oral medication for chronic HF according to GDMT and without signs of fluid overload or low cardiac output were included. Reasons for ineligibility included significant anemia (Hb <11 g/dL), utilizing erythropoiesis stimulation agents or having used oral iron supplementation or intravenous iron treatment within the last 3 months. Individuals with a planned resynchronization therapy, coronary revascularization, heart transplant or left ventricular assistance device implantation or with less than one year of life expectancy were also excluded.

**Study procedures.** Patients were classified according to the current ESC guideline definition of ID (serum ferritin <100 ug/L or ferritin 100–299 ug/L with TSAT <20%) [16]. ID patents were treated following clinical recommendations; the dosage was calculated according to their weight and hemoglobin levels. Ferric carboxymaltose (FCM, Ferinject, CSL Vifor) was administered as an intravenous perfusion diluted in a sterile solution administered over 30–60 min. The patients received a maximum of 1000 mg of iron all at once. In patients for whom more iron was needed, a second dose was administered, at least fifteen days after the first one.

**Study data.** A detailed baseline evaluation was performed for all participants at study entry. Demographic characteristics, clinical and disease-related factors, comorbidities, laboratory tests and medical treatments were collected. Patients with ID were re-assessed after 3 months of iron replenishment (a clinical evaluation, a blood test and an echocardiography were performed). Clinical events were evaluated on an ongoing basis in all patients and finally ascertained after 12 months of follow-up.

**Echocardiographic evaluation.** Echocardiographic assessment was performed at baseline. In the ID group, echocardiography was repeated 3 months after intravenous iron replacement. Echocardiographic examinations were performed by two experienced research sonographers blinded to the iron status of the patient and the therapeutic interventions conducted in terms of iron repletion. Standard commercial Vivid E97 equipment and an MC5 active-matrix transducer (GE Medical Systems, Chicago, IL, USA) were used. All the images were digitally stored online and measured with specific offline software (EchoPAC Version 202, GE Medical Systems). All images were acquired throughout a complete cycle at a frame rate that represents 80% of the heart rate without exceeding 90 Hz or falling below 40 Hz. The data were transferred for further analysis in specific software. Measurements were made in three cycles (five cycles in patients that received atrial fibrillation during the examination), and the average value was calculated.

The echocardiographic features included the bidimensional and diastolic parameters (septal wall, posterior wall, LV end-diastolic diameter, LV end-diastolic volume, LA indexed volume, E/A ratio, E/e′ ratio), left and right ventricle function parameters (LVEF, GLS, LV indexed stroke volume, LV indexed stroke work, cardiac output, TAPSE, systolic pulmonary artery pressure, FAC, RV coupling ratio, RV free wall strain) and vascular function parameters (systemic arterial compliance, systemic arterial resistance index, LV end-systolic elastance (Ees), arterial elastance (Ea), ventricular–arterial coupling) (Table S1).

MW was assessed via the combination of LV strain data, the non-invasively estimated LV pressure curve (brachial cuff blood pressure) and the valvular event timing [14]. Strain and pressure data were synchronized using the onset of the R-wave in EKG, and the area of the pressure strain loop was used to derive the segmental and global MW. The global work index (GWI) was calculated as the average of segmental values. The CW was defined as work during segmental shortening in systole and during lengthening in isovolumic relaxation. WW (energy loss) is the work performed during lengthening in systole and shortening in isovolumic relaxation. Work efficacy (WE) was automatically calculated as the ratio of CW/(CW + WW).

**Study outcomes**. The main endpoint was the change in cardiac performance, including LVEF and LV deformation parameters (GLS and its derivates MW, WW, CW and WE) after iron replenishment. Secondary endpoints included the change in RV function parameters (TAPSE, FAC, RV free wall strain and RV coupling ratio). Finally, exploratory endpoints included cardiovascular function explored by means of Ea, Ees and VAC.

**Statistical analysis**. Variables are expressed as the mean and standard deviations (in the case of normal distribution) or median and interquartile ranges (in the case of non-normal distribution). Boxplots were used to graphically describe the distribution of the variables. A comparison was performed using the chi-squared test (for qualitative variables), the *t*-test (for quantitative variables with normal distribution) or the Mann–Whitney-U–Wilcoxon (for quantitative variables with non-normal distribution). The *t*-test was used for the variables that met the assumptions of independence, normality (using the Kolmogorov–Smirnov test) and homoscedasticity or equality of variances (using the Levene test).

The comparison between the baseline echocardiographic parameters (before iron replenishment) and those 3 months after iron replenishment was performed using a *t*-test for paired data. The 3-month variables of the iron-deficient group (after iron replenishment) were compared with the baseline variables of the non-iron-deficient group. Bar graphs were used to graphically describe the evolution of the variables. No multivariate statistical model was used.

All statistical tests and confidence intervals (CIs) were constructed with a Type I error, alpha level of 5%, with no adjustments for multiplicity. *p*-values below 0.05 were considered statistically significant. All analyses were performed using SPSS software (version 22.0; IBM, Armonk, NY, USA) and R software packages (version 4.2.1; R Foundation for Statistical Computing, Vienna, Austria).

## 3. Results

The IRON-PATH II echo-substudy included a subset of 100 patients undergoing a specific echocardiographic evaluation. Two patients were excluded due to a poor sonographic window. Thus, 98 patients were finally analyzed (Figure S1).

### 3.1. Baseline Characteristics

The baseline characteristics of the whole IRON-PATH II study cohort are presented in Table S2. The patients included in the echo-substudy cohort were slightly older (72 vs. 68 years, *p* = 0.027) and had worse renal function (eGFR 55 vs. 60 mL/min/1.73 m^2^, *p* = 0.044). Ferritin levels were lower in patients not included in the echo-substudy cohort (ferritin 244 vs. 138 ng/mL, *p* < 0.001) even though no differences in TSAT were observed (22 vs. 21%, *p* = 0.351).

The baseline characteristics of the echo-substudy sample, both overall and according to ID status, are listed in Table 1. ID was present in 44 patients (45%). Considering the clinical profile, ID patients were older (74 ± 8 vs. 70 ± 11 years; *p*-value = 0.03), more frequently male (78%), NYHA class II (71%) and had a slightly higher heart rate (72 vs. 67 bpm, *p* = 0.043). They also covered shorter distances in the 6 min walking distance (206 vs. 314 m, *p* < 0.001). Comorbidities appeared to be similar in the two groups, with the exception of atrial fibrillation (28 vs. 22%, *p*-value = 0.042).

The mean hemoglobin levels were lower among ID patients (13.2 vs. 14.2, *p* = 0.001), and the NTproBNP levels were higher (2180 vs. 997 pg/mL, *p*-value = 0.015). The ID group showed marked iron depletion evidenced by lower transferrin saturation (TSAT) (16 vs. 28%), ferritin (116 vs. 349 ng/mL) and iron (9 vs. 15 µmol/L) (all *p*-values < 0.001). Both groups were similarly treated (Table 1) with beta-blockers, angiotensin receptor neprilysin inhibitors (ARNIs), sodium–glucose cotransporter-2 (SGLT2i) and mineralocorticoid receptor antagonist (MRA) (94%, 76%, 71% and 76%, respectively), all *p*-values > 0.05. Anticoagulation therapy was more frequent among the ID patients (46% vs. 73%, *p*-value = 0.021).

### 3.2. Echocardiographic Features at Baseline

The groups according to ID status showed no differences relative to LV thickness, LV volumes, E/A ratio or E/e’ ratio (all *p*-values > 0.05) (Table 2). No differences were observed in the LVEF (38 ± 10% vs. 35 ± 9%, *p*-value = 0.210), TAPSE (18 mm vs. 17 mm, *p*-value = 0.118) or sPAP (29 mmHg vs. 30 mmHg, *p*-value = 0.150). The ID group displayed worse LV function expressed by lower GLS (−8.5 ± 9% vs. −10 ± 10%, *p*-value = 0.024), lower work efficiency (74 ± 10% vs. 80 ± 10%, *p*-value = 0.017), lower myocardial work (665 [453–1013] mmHg% vs. 947 [542–1199] mmHg%, p-value = 0.025) and higher wasted work (290 [228–384] mmHg% vs. 212 [138–305] mmHg%, *p*-value = 0.034) (Table 3 and Figure S2, panel A). The RV function was also worse, as shown by a lower RV free wall strain (−13 [−20–(−11)] % vs. −17 [−23–(−14)]%, *p*-value = 0.022) (Table 3 and Figure S2, panel B). However, no differences were observed regarding TAPSE (18 vs. 17 mm, *p*-value = 0.118) or sPAP (29 [24–35] vs. 30 [26–42] mmHg, *p*-value = 0.15). Finally, the ID group obtained worse end-diastolic elastance values (3.0 vs. 2.1 mmHg/mL, *p*-value = 0.03) and a worse ventricle–arterial coupling ratio (0.95 vs. 1.4 mmHg/mL, *p*-value = 0.04) (Table 3 and Figure S2, panel C).

### 3.3. Echocardiographic Features After Iron Repletion

**Figure 1:**
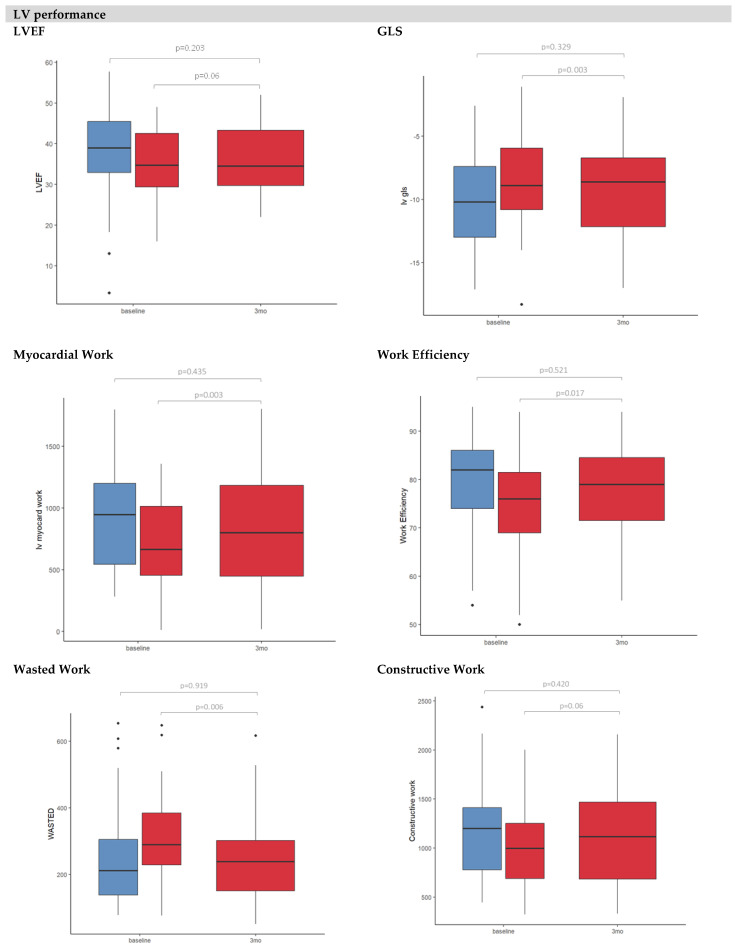

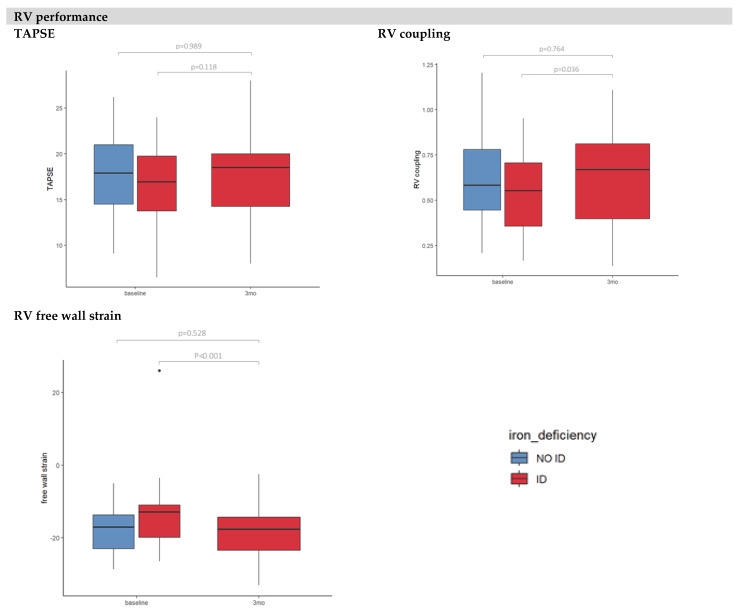
Bar graphs (showing mean and standard deviations) of LV myocardial performance (LVEF, GLS, myocardial work, constructive work, wasted work and work efficiency) and RV myocardial performance (TAPSE, RV coupling, FAC, RV free wall strain). No ID (blue boxes) vs. ID (red boxes).

After intravenous iron replacement (Table 3), improvements in GLS (−8.5 ± 9% vs. −9.3 ± 3%), work efficiency (74 ± 10% vs. 79 ± 10%), myocardial work (665 [453–1013] mmHg% vs. 801 [447–1183] mmHg%) and wasted work (290 [228–384] mmHg% vs. 239 [151–302] mmHg%) were observed (all *p*-values <0.05). Regarding the RV function, patients had an improved RV coupling ratio (0.54 ± 2 vs. 0.61 ± 0.3 mm/mmHg, *p*-value = 0.036) and RV free wall strain (−13 [−20 to −11]% vs. −18 [−24 to −14]%, *p*-value <0.001). Iron replacement had no impact on LV end-diastolic elastance or ventricle–arterial coupling (Ees/Ea). No differences were observed in any echocardiographic parameter of myocardial function between non-ID patients in comparison with ID-patients after iron replenishment (Figure 1 and Table 3).

## 4. Discussion

Our study provides relevant information regarding the pathophysiology of ID in the HF and LVEF <50% scenario. Firstly, patients with HF and ID displayed worse myocardial performance than non-ID patients, for both LV (GLS, myocardial work, wasted work and work efficiency) and RV (RV free wall strain and RV coupling), with no differences in LVEF or TAPSE. Secondly, iron repletion significantly improved myocardial performance in ID patients, resulting in a myocardial performance phenotype similar to that in non-ID patients.

There is accumulating evidence that ID is not only a consequence but also a contributor to the pathophysiology of systolic HF that potentially exacerbates disease progression through the mitochondrial dysfunction caused by intracellular iron depletion [17,18,19]. Previous studies showed that correcting anemia and ID results in an improvement in echocardiographic parameters, suggesting the role of these conditions in myocardial mechanics [5]. Toblli et al. demonstrated an enhancement in the LVEF (6.6 ± 3.8%) and a reduction in LV diameters (LVESD 10.5 ± 1.6 mm, LVEDD 9 ± 13 mm) with iron sucrose therapy among HF with reduced LVEF patients [20]. The IRON-CRT study [21] included 75 patients with HF, ID criteria and an LVEF of 45% or less. After CRT therapy, a greater increase in LVEF in response to CRT was observed in the group in which FCM was replaced when compared to placebo. The response was analyzed 3 months after iron replacement. The improvement was due to LV end-systolic volume (LVESV) reduction, with no differences in the LV end-diastolic volume (LVEDV).

Little evidence exists regarding the effects of iron repletion on strain parameters [11]. To our knowledge, only the Myocardial-IRON substudy [22] has explored the effects of iron repletion on myocardial function expressed through strain parameters. Improvement in the global longitudinal, circumferential and radial strain of both the LV and RV was observed 30 days after iron repletion. In COPD patients without anemia, ID was associated with a moderate increase in systolic pulmonary artery pressure [23]. However, no work to date has assessed the right ventricular remodeling associated with iron deficiency. Thus, in patients with HF, the impact of iron replacement on RV has not been adequately explored.

*Myocardial work as a surrogate of cardiac function, back to the pressure–volume loop.* Our cohorts showed no differences in the LVEF, LV stroke volume, LV stroke work, TAPSE or sPAP (all *p*-values >0.05). Nevertheless, in the IRON-PATH II echo-substudy, we employed speckle-tracking echocardiography (GLS, myocardial work index and its derivates) and VAC assessment as myocardial performance subrogates. These tools offer significant advantages over traditional measures like the LVEF as they offer a more accurate assessment of myocardial function by integrating both cardiac and arterial components [13,24,25]. The analysis of pressure–volume loops allows for the measurement of the energy imparted to the blood, with the area of the loop representing stroke work (SW) [26]. New echocardiography software creates arterial pressure–LV longitudinal myocardial strain curves by speckle-tracking [14]. This software calculates myocardial work, constructive work (work during segmental shortening in systole and during lengthening in isovolumic relaxation) and waste work (lengthening in systole and shortening in isovolumic relaxation).

Non-invasive methods for assessing myocardial work have shown good agreement with invasive pressure–volume loop measurements [27]. Thus, the myocardial work index allows for a comprehensive assessment of the energy dynamics within the heart while considering the loading conditions. In addition to the myocardial work index (work evaluated from mitral valve closure valve opening), waste work is calculated as the myocardial work consumed during segmental lengthening (negative work) that does not turn into segmental contraction (positive work) [14,27].

These parameters have been previously explored in the HF scenario. Sacubitril–Valsartan treatment has been shown to improve constructive work and work efficiency [28]; likewise, these two parameters have been proposed as a predictor of events in advanced HF [29] and as a predictor index of CRT response [30]. To the best of our knowledge, this is the first study demonstrating myocardial work improvement after iron repletion, due to a decrease in waste work and an improvement in work efficiency. The RV functional parameters like the RV coupling ratio and RV free wall strain also improved. Interestingly, these functional parameters were similar to those seen in the non-ID group after iron repletion.

Beyond myocardial performance, VAC makes it possible to study the relationship between the myocardium and the arterial tree. VAC is evaluated by non-invasively measuring the ratio of arterial (Ea) to ventricular end-systolic elastance (Ees) [31,32]. Ea is an integrative measure of arterial system properties [33], while Ees defines the ventricle’s contractile state and is relatively insensitive to loading conditions. Thus, VAC evaluation together with the interpretation of its components (Ea and Ees) allows for a more complete myocardial performance profile in patients with heart failure (HF) and iron deficiency (ID).

Ventricle–arterial uncoupling in HFrEF is characterized by a decrease in Ees together with an increase in Ea (derived from neurohormonal activation), which leads to higher VAC values [31]. Either CRT implantation [34] or pharmacological treatment with a β-blocker [35] may improve VAC. Patients with ID showed worse VAC at the expense of a lower Ees. However, no differences in Ees, Ea or VAC were observed after iron replacement.

*The role of iron in myocardial performance*. Myocardial activity is closely linked to oxidative metabolism [36]. Notably, not all the energy generated by oxidative metabolism is utilized and converted into effective work. Therefore, our data suggests that ID may contribute to HF progression based on the lower myocardial work (likely related to the higher waste work displayed by ID patients) and reversibility seen after iron replacement.

Clinically, the iron-deficient group was probably composed of patients with a true iron-deficient state, as reflected by a mean TSAT of 16%. The significant improvements observed in our study can be explained by the restoration of mitochondrial function and energy production in cardiomyocytes due to iron repletion [37]. Notably, ID repletion had a positive effect on cardiac performance even though AF was more frequent and the mean left atria was larger in patients in the ID group. Finally, the positive changes in RV function and ventricular–arterial coupling suggest that iron therapy may also improve pulmonary hemodynamics and right ventricular performance, thereby contributing to better overall heart function.

The ID group showed significantly higher NTproBNP levels (997 vs. 2180 ng/L, *p* = 0.015). The improvement in myocardial performance was not linked to a change in clinical congestion, since NTproBNP levels did not decrease in the control at 3 months (2180 vs. 2260 ng/L, *p* = 0.34). All patients included were in a clinically stable situation with no signs or symptoms of hypervolemia. The elevated NTproBNP values were probably related to subclinical congestion and worse iron absorption.

Our study highlights the significant improvements in myocardial performance following intravenous iron repletion in patients with HF and ID. These findings underscore the potential of iron therapy to enhance cardiac function beyond traditional measures such as the LVEF or TAPSE. Future research should include mechanistic studies to shed light on the pathways through which medical therapies, in general, and iron repletion, in particular, benefit myocardial function. These insights will pave the way for more targeted and effective therapeutic strategies.

**Limitations.** Our study has some limitations that should be mentioned. First, our study is based on echocardiography. Therefore, the performance of cMRI in the study of myocardial work after iron replacement is uncertain. However, echocardiography is an accessible technique, and myocardial work calculation is an easy-to-use and reproducible tool as it has been included in echocardiographic software. Second, speckle-tracking requires experience and depends on image quality. Non-invasive arterial pressure is included in the calculation of myocardial work, which can be variable throughout the cardiac cycle. Third, both groups were well balanced. However, ID patients were slightly older and had a higher incidence of AF and lower mean hemoglobin levels. Although this may partially explain the baseline differences, the improvement in myocardial performance after iron replacement was clear. The echocardiographic evaluation was performed 3 months after iron replenishment. However, the duration of the potential beneficial effect on myocardial performance is unknown. Finally, both groups underwent baseline echocardiography, but only the ID group received control echocardiography (3 months after replacement). Thus, direct comparison between the two groups is limited.

## 5. Conclusions

The IRON-PATH II echo-substudy demonstrates that HF patients with a reduced LVEF and ID have worse myocardial performance in both the LV and RV. Specifically, ID patients had impaired GLS, work efficiency, myocardial work, waste work, RV free wall strain and RV coupling. No differences were observed in terms of the LVEF or TAPSE. These alterations in myocardial function were measurable via echocardiography and were reversible with the intravenous repletion of ferric carboxymaltose. This underscores the significant role of iron therapy in enhancing cardiac function. Future research should explore the underlying mechanisms and further validate these findings to optimize therapeutic strategies.

## Figures and Tables

**Table 1:** Demographic and clinical characteristics of all patients included in this analysis, overall and according to iron status.

	Whole Cohort (n = 98)	No ID (n = 54)	ID (n = 44)	*p*-Value
**Demographics**				
Age, years	72 (10)	70 (11)	74 (8)	0.030
Sex (female), n (%)	22 (22)	16 (30)	6 (14)	0.059
Systolic blood pressure, mmHg	119 (19)	118 (21)	119 (18)	0.627
Heart rate, bpm	69 (12)	67 (11)	72 (13)	0.043
NYHA functional class, n (%)				0.095
I	16 (16)	12 (22)	4 (9)	
II	70 (71%)	38 (71%)	32 (72%)	
III	10 (10%)	3 (6%)	7 (16%)	
IV	2 (2%)	0 (0%)	2 (4%)	
6 min walking test, meters	357 (102)	366 (108)	346 (86)	0.359
BMI, kg/m^2^	27 (4)	27 (4)	27 (5)	0.423
CV hospitalization in previous year, n (%)	45 (46%)	21 (21%)	24 (25%)	0.311
**Comorbidities**				
Ischemic etiology of HF, n (%)	49 (50%)	20 (20%)	29 (30%)	0.103
Hypertension, n (%)	76 (78%)	39 (40%)	37 (38%)	0.161
Diabetes mellitus, n (%)	47 (48%)	22 (22%)	25 (26%)	0.189
Previous MI, n (%)	50 (51%)	23 (24%)	27 (28%)	0.064
PAD, n (%)	15 (15%)	9 (9%)	6 (6%)	0.679
Atrial fibrillation, n (%)	49 (50%)	22 (22%)	27 (28%)	0.042
**Laboratory**				
Hemoglobin, g/dL	14.1 (1.4)	14.2 (1.5)	13.2 (1.3)	0.001
Creatinine, umol/L	124.7 (52.7)	123.2 (51.1)	126.4 (55.2)	0.771
Estimated glomerular filtration rate, mL/min/kg	55 (21)	56 (21)	54 (21)	0.709
Sodium, mmol/L	141 (2)	141 (2)	141 (3)	0.336
Potassium, mmol/L	4.75 (0.43)	4.72 (0.40)	4.78 (0.47)	0.554
Ferritin, ng/mL	244 (204)	349 (216)	116 (73)	<0.001
TSAT, %	22 (9)	28 (7)	16 (6)	<0.001
Iron, umol/L	13 (5)	15 (4)	9 (4)	<0.001
Transferrin, umol/L	32.8 (30.2)	32.4 (37.2)	33.3 (18.8)	0.890
TIBC, umol/L	58 (9.7)	55.5 (8.3)	61.2 (10.3)	0.003
NT-proBNP, pg/mL (median, IQR)	1793 (697–3381)	997 (510–3098)	2180 (1226–3698)	0.015
**Treatment**				
ARNI, n (%)	74 (76%)	42 (78%)	32 (73%)	0.563
ACEI or ARBs, n (%)	12 (12%)	5 (9%)	7 (16%)	0.732
Beta-blockers, n (%)	92 (94%)	50 (93%)	42 (95%)	0.557
MRA, n (%)	74 (76%)	42 (78%)	32 (73%)	0.563
iSGLT2, n (%)	70 (71%)	37 (68%)	33 (75%)	0.674
Diuretics, n (%)	73 (75%)	35 (65%)	38 (86%)	0.046
Antiplatelet therapy, n (%)	38 (39%)	23 (42%)	15 (34%)	0.390
Anticoagulant therapy, n (%)	57 (57%)	25 (46%)	32 (73%)	0.021
Cardiac resynchronization therapy, n (%)	9 (9%)	4 (7%)	5 (11%)	0.500
Implantable cardioverter defibrillator device, n (%)	15 (15%)	8 (15%)	7 (16%)	0.881

**Table 2:** Echocardiographic characteristics of all patients included in this analysis, overall and according to tissue iron status.

	Whole Cohort (n = 98)	No ID (n = 54)	ID (n = 44)	*p*-Value
Septal wall, mm	11 (3)	11 (3)	11 (3)	0.890
Posterior wall, mm	10 (2)	10 (2)	10 (2)	0.267
LV end-diastolic diameter, mm	55 (10)	55 (10)	55 (10)	0.803
LV indexed end-diastolic volume, mL/m^2^	78 (31)	76 (32)	80 (28)	0.452
LV indexed end-systolic volume, mL/m^2^	50 (26)	46 (27)	54 (25)	0.182
LA indexed volume, mL/m^2^	49 (17)	45 (17)	53 (16)	0.026
E/A ratio	1.4 (1)	1.3 (1)	1.5 (1)	0.661
E/e′ ratio	13 (7)	13 (6)	14 (9)	0.596
LVEF, %	36 (9.66)	38 (10.26)	35 (8.78)	0.210
TAPSE, mm	17 (4.18)	18 (4.24)	17 (4.03)	0.118
Systolic pulmonary artery pressure, mmHg (median, IQR)	30 (25–37)	29 (24–35)	30 (26–42)	0.15

LV: left ventricle. LVEF: left ventricle ejection fraction. TAPSE: tricuspid annular plane systolic excursion.

**Table 3:** *T*-test and Mann–Whitney-U exploring echocardiographic characteristics differences before (ID baseline) and after (ID 3rd month) intravenous iron replacement (in ID group), as well as after iron replacement (ID 3rd month) versus non-ID group.

	Whole Cohort (n = 98)	Non-ID	ID Baseline Before Iron Replacement	ID 3rd Month After Iron Replacement	*p*-Value ID Baseline vs. Non-ID Baseline	*p*-Value ID Baseline vs. ID 3rd Month	*p*-Value ID 3rd Month vs. Non-ID
**Left ventricular function**							
LVEF, %	36 (9.66)	38 (10.26)	35 (8.78)	36 (9.66)	0.210	0.06	0.203
GLS, %	−9.5 (3.69)	−10.2 (10.26)	−8.5 (8.78)	−9.31 (3.43)	0.024	0.003	0.329
LV indexed stroke work, g.m/m^2^	33 (13)	34 (9)	32 (17)	31 (10)	0.423	0.422	0.564
Cardiac output, L/min (median, IQR)	4.7 (3.8–5.7)	4.7 (3.78–5.90)	4.61 (3.93–5.46)	4.2 (3.50–4.40)	0.62	0.08	0.131
Cardiac index, L/min/m^2^ (median, IQR)	2.4 (2–2.5)	2.6 (2.1–3)	2.7 (2.5–2.9)	2.3 (2–2.7)	0.728	0.075	0.135
Myocardial work, mmHg% (median, IQR)	823 (504–1113)	947 (542–1199)	665 (453–1013)	801 (447–1183)	0.025	0.003	0.435
Constructive work, mmHg%	1131 (449.10)	1191 (449.82)	1053 (441.63)	1108(471)	0.149	0.06	0.420
Wasted work, mmHg% (median, IQR)	262 (155–361)	212 (138–305)	290 (228–384)	239 (151–302)	0.034	0.006	0.919
Work efficiency, %	77.41 (10.37)	80 (9.93)	74 (10.32)	79 (9.98)	0.017	0.017	0.521
**Right ventricular function**							
TAPSE, mm	17 (4.18)	18 (4.24)	17 (4.03)	17 (4.31)	0.118	0.118	0.989
Systolic pulmonary artery pressure, mmHg (median, IQR)	30 (25–37)	29 (24–35)	30 (26–42)	28.10 (24.72–40.28)	0.15	0.58	0.73
FAC, % (median, IQR)	45 (38–52)	45.45 (38.74–54.69)	45.19 (36.93–49.66)	44 (35.45–47.57)	0.095	0.68	0.162
RV coupling ratio, mm/mmHg	0.59 (0.23)	0.63 (0.25)	0.54 (0.21)	0.61 (0.27)	0.083	0.036	0.764
RV free wall strain, % (median, IQR)	−16 (-22–−12)	−17 (-23–−14)	−13 (-20–−11)	−17.6 (−23.5–−14.3)	0.022	<0.001	0.528
**Cardiovascular function**							
Systemic arterial compliance, mL/m^2^/mmHg (median, IQR)	1.3 (1–1.6)	1.26 (1.01–1.56)	1.27 (1.04–1.63)	1.1 (0.90–1.51)	0.93	0.16	0.09
Systemic arterial resistance index, dyn·s·cm^−5^·m^−2^	2449 (764.39)	2442 (751)	2458 (789)	2690 (822)	0.923	0.10	0.217
LV end-systolic elastance, mmHg/mL (median, IQR)	2.5 (1.7–3.5)	3.0 (1.9–3.6)	2.1 (1.5–3.1)	2.34 (1.53–3.42)	0.03	0.20	0.511
Arterial elastance, mmHg/mL (median, IQR)	2.8 (2.4–3.4)	2.7 (2.4–3.3)	3.0 (2.4–3.7)	3.26 (2.73–3.80)	0.26	0.1	0.011
Ventricular–arterial coupling (ratio) (median, IQR)	1.1 (0.8–1.9)	0.95 (0.75–1.4)	1.4 (0.9–2.2)	1.46 (0.86–2.37)	0.04	0.53	0.109

LV: left ventricle. LVEF: left ventricle ejection fraction. GLS: global longitudinal strain. TAPSE: tricuspid annular plane systolic excursion. FAC: fractional area change. RV: right ventricle.

## Data Availability

Data is contained within this article or Supplementary Materials.

## Supplementary Materials

The following supporting information can be downloaded at https://www.mdpi.com/article/10.3390/jcm14124048/s1, Table S1: LV end-systolic elastance (Ees), arterial elastance (Ea) and ventricular–arterial coupling formulas and normal values. Table S2: Demographic and clinical characteristics of all patients included in this analysis, overall and according to iron status. Figure S1: Flowchart. Figure S2: Boxplots (showing mean and standard deviations) of LV myocardial performance (LVEF, GLS, myocardial work, constructive work, wasted work and work efficiency), RV myocardial performance (TAPSE, RV coupling, FAC, RV free wall strain) and ventricular–arterial coupling (systemic arterial resistance, LV elastance, arterial elastance and VAC) according to iron status.

## Abbreviations

The following abbreviations are used in this manuscript:

AF	Atrial fibrillation
ARNI	Angiotensin receptor neprilysin inhibitor
ATP	Adenosine triphosphate
CRT	Cardiac resynchronization therapy
CW	Constructive work
Ea	Arterial elastance
Ees	End-systolic elastance
ESC	European Society of Cardiology
FCM	Ferric carboxymaltose
FAC	Fractional area change
GLS	Global longitudinal strain
GWI	Global work index
Hb	Hemoglobin
HF	Heart failure
ID	Iron deficiency
LVEF	Left ventricular ejection fraction
LVEDV	Left ventricular end-diastolic volume
LVESD	Left ventricular end-systolic diameter
LVESV	Left ventricular end-systolic volume
MW	Myocardial work
MRA	Mineralocorticoid receptor antagonist
NTproBNP	N-terminal pro-brain natriuretic peptide
NYHA	New York Heart Association
RV	Right ventricle
RVFW	Right ventricular free wall
sPAP	Systolic pulmonary artery pressure
TAPSE	Tricuspid annular plane systolic excursion
TSAT	Transferrin saturation
VAC	Ventricular–arterial coupling
WE	Work efficiency
WW	Wasted work

## References

[1] Melenovsky V., Petrak J., Mracek T., Benes J., Borlaug B.A., Nusková H., Pluhacek T., Spatenka J., Kovalcikova J., Drahota Z. (2017). Myocardial iron content and mitochondrial function in human heart failure: A direct tissue analysis. Eur. J. Heart Fail..

[2] Rocha B.M.L., Cunha G.J.L., Falcão L.F. (2018). The Burden of Iron Deficiency in Heart Failure. J. Am. Coll. Cardiol..

[3] Hoes M.F., Grote Beverborg N., Kijlstra J.D., Kuipers J., Swinkels D.W., Giepmans B.N.G., Rodenburg R.J., Van Veldhuisen D.J., De Boer R.A., Van Der Meer P. (2018). Iron deficiency impairs contractility of human cardiomyocytes through decreased mitochondrial function. Eur. J. Heart Fail..

[4] Zhang H., Jamieson K.L., Grenier J., Nikhanj A., Tang Z., Wang F., Wang S., Seidman J.G., Seidman C.E., Thompson R. (2022). Myocardial Iron Deficiency and Mitochondrial Dysfunction in Advanced Heart Failure in Humans. J. Am. Heart Assoc..

[5] Sutil-Vega M., Rizzo M., Martínez-Rubio A. (2019). Anemia and iron deficiency in heart failure: A review of echocardiographic features. Echocardiography.

[6] Graham F.J., Pellicori P., Kalra P.R., Ford I., Bruzzese D., Cleland J.G.F. (2023). Intravenous iron in patients with heart failure and iron deficiency: An updated meta-analysis. Eur. J. Heart Fail..

[7] Anker S.D., Comin Colet J., Filippatos G., Willenheimer R., Dickstein K., Drexler H., Lüscher T.F., Bart B., Banasiak W., Niegowska J. (2009). Ferric Carboxymaltose in Patients with Heart Failure and Iron Deficiency. N. Engl. J. Med..

[8] Ponikowski P., Kirwan B.-A., Anker S.D., McDonagh T., Dorobantu M., Drozdz J., Fabien V., Filippatos G., Göhring U.M., Keren A. (2020). Ferric carboxymaltose for iron deficiency at discharge after acute heart failure: A multicentre, double-blind, randomised, controlled trial. Lancet.

[9] Jankowska E.A., Kirwan B.-A., Kosiborod M., Butler J., Anker S.D., McDonagh T., Dorobantu M., Drozdz J., Filippatos G., Keren A. (2021). The effect of intravenous ferric carboxymaltose on health-related quality of life in iron-deficient patients with acute heart failure: The results of the AFFIRM-AHF study. Eur. Heart J..

[10] Kalra P.R., Cleland J.G.F., Petrie M.C., A Thomson E., A Kalra P., Squire I.B., Ahmed F.Z., Al-Mohammad A., Cowburn P.J., Foley P.W.X. (2022). Intravenous ferric derisomaltose in patients with heart failure and iron deficiency in the UK (IRONMAN): An investigator-initiated, prospective, randomised, open-label, blinded-endpoint trial. Lancet.

[11] Gaber R., Kotb N.A., Ghazy M., Nagy H.M., Salama M., Elhendy A. (2012). Tissue Doppler and Strain Rate Imaging Detect Improvement of Myocardial Function in Iron Deficient Patients with Congestive Heart Failure after Iron Replacement Therapy. Echocardiography.

[12] Marwick T.H. (2018). Ejection Fraction Pros and Cons. J. Am. Coll. Cardiol..

[13] Brady B., King G., Murphy R.T., Walsh D. (2023). Myocardial strain: A clinical review. Ir. J. Med. Sci. (1971).

[14] Ilardi F., D’Andrea A., D’Ascenzi F., Bandera F., Benfari G., Esposito R., Malagoli A., Mandoli G.E., Santoro C., Russo V. (2021). Myocardial Work by Echocardiography: Principles and Applications in Clinical Practice. J. Clin. Med..

[15] Enjuanes C., Bruguera J., Grau M., Cladellas M., Gonzalez G., Meroño O., Moliner-Borja P., Verdú J.M., Farré N., Comín-Colet J. (2016). Estado del hierro en la insuficiencia cardiaca crónica: Impacto en síntomas, clase funcional y capacidad de ejercicio submáxima. Rev. Esp. Cardiol..

[16] McDonagh T.A., Metra M., Adamo M., Gardner R.S., Baumbach A., Böhm M., Burri H., Butler J., Čelutkienė J., Chioncel O. (2021). 2021 ESC Guidelines for the diagnosis and treatment of acute and chronic heart failure. Eur. Heart J..

[17] Tajes M., Díez-López C., Enjuanes C., Moliner P., Ferreiro J.L., Garay A., Jiménez-Marrero S., Yun S., Sosa S.G., Alcoberro L. (2021). Neurohormonal activation induces intracellular iron deficiency and mitochondrial dysfunction in cardiac cells. Cell Biosci..

[18] Díez-López C., Orduña M.T., Grau C.E., Borja P.M., González-Costello J., García-Romero E., Manzano J.F., Viladomat S.Y., Jiménez-Marrero S., Ramos-Polo R. (2021). Blood Differential Gene Expression in Patients with Chronic Heart Failure and Systemic Iron Deficiency: Pathways Involved in Pathophysiology and Impact on Clinical Outcomes. J. Clin. Med..

[19] Alnuwaysir R.I.S., Hoes M.F., van Veldhuisen D.J., van der Meer P., Beverborg N.G. (2021). Iron Deficiency in Heart Failure: Mechanisms and Pathophysiology. J. Clin. Med..

[20] Toblli J.E., Di Gennaro F., Rivas C. (2015). Changes in Echocardiographic Parameters in Iron Deficiency Patients with Heart Failure and Chronic Kidney Disease Treated with Intravenous Iron. Heart Lung Circ..

[21] Martens P., Dupont M., Dauw J., Nijst P., Herbots L., Dendale P., Vandervoort P., Bruckers L., Tang W.H.W., Mullens W. (2021). The effect of intravenous ferric carboxymaltose on cardiac reverse remodelling following cardiac resynchronization therapy—The IRON-CRT trial. Eur. Heart J..

[22] Del Canto I., Santas E., Cardells I., Miñana G., Palau P., Llàcer P., López-Vilella R., Almenar L., Bodí V., López-Lereu M.P. (2022). Short-Term Changes in Left and Right Ventricular Cardiac Magnetic Resonance Feature Tracking Strain Following Ferric Carboxymaltose in Patients With Heart Failure: A Substudy of the Myocardial-IRON Trial. J. Am. Heart Assoc..

[23] Plesner L.L., Schoos M.M., Dalsgaard M., Goetze J.P., Kjøller E., Vestbo J., Iversen K. (2017). Iron Deficiency in COPD Associates with Increased Pulmonary Artery Pressure Estimated by Echocardiography. Heart Lung Circ..

[24] Paolisso P., Gallinoro E., Mileva N., Moya A., Fabbricatore D., Esposito G., De Colle C., Beles M., Spapen J., Heggermont W. (2022). Performance of non-invasive myocardial work to predict the first hospitalization for de novo heart failure with preserved ejection fraction. ESC Heart Fail..

[25] Nikoo M.H., Naeemi R., Moaref A., Attar A. (2020). Global longitudinal strain for prediction of ventricular arrhythmia in patients with heart failure. ESC Heart Fail..

[26] Monge García M.I., Santos A. (2020). Understanding ventriculo-arterial coupling. Ann. Transl. Med..

[27] Russell K., Eriksen M., Aaberge L., Wilhelmsen N., Skulstad H., Remme E.W., Haugaa K.H., Opdahl A., Fjeld J.G., Gjesdal O. (2012). A novel clinical method for quantification of regional left ventricular pressure–strain loop area: A non-invasive index of myocardial work. Eur. Heart J..

[28] Bouali Y., Donal E., Gallard A., Laurin C., Hubert A., Bidaut A., Leclercq C., Galli E. (2020). Prognostic Usefulness of Myocardial Work in Patients with Heart Failure and Reduced Ejection Fraction Treated by Sacubitril/Valsartan. Am. J. Cardiol..

[29] Hedwig F., Nemchyna O., Stein J., Knosalla C., Merke N., Knebel F., Hagendorff A., Schoenrath F., Falk V., Knierim J. (2021). Myocardial Work Assessment for the Prediction of Prognosis in Advanced Heart Failure. Front. Cardiovasc. Med..

[30] Galli E., Leclercq C., Hubert A., Bernard A., A Smiseth O., Mabo P., Samset E., Hernandez A., Donal E. (2018). Role of myocardial constructive work in the identification of responders to CRT. Eur. Heart J. Cardiovasc. Imaging.

[31] Ikonomidis I., Aboyans V., Blacher J., Brodmann M., Brutsaert D.L., Chirinos J.A., De Carlo M., Delgado V., Lancellotti P., Lekakis J. (2019). The role of ventricular–arterial coupling in cardiac disease and heart failure: Assessment, clinical implications and therapeutic interventions. A consensus document of the European Society of Cardiology Working Group on Aorta & Peripheral Vascular Diseases, European Association of Cardiovascular Imaging, and Heart Failure Association. Eur. J. Heart Fail..

[32] Chen C.H., Fetics B., Nevo E., Rochitte C.E., Chiou K.-R., Ding P.A., Kawaguchi M., Kass D.A. (2001). Noninvasive single-beat determination of left ventricular end-systolic elastance in humans. J. Am. Coll. Cardiol..

[33] Chirinos J.A., Sweitzer N. (2017). Ventricular–Arterial Coupling in Chronic Heart Failure. Card. Fail. Rev..

[34] Zanon F., Aggio S., Baracca E., Pastore G., Corbucci G., Boaretto G., Braggion G., Piergentili C., Rigatelli G., Roncon L. (2009). Ventricular-arterial coupling in patients with heart failure treated with cardiac resynchronization therapy: May we predict the long-term clinical response?. Eur. J. Echocardiogr..

[35] Dekleva M., Lazic J.S., Soldatovic I., Inkrot S., Arandjelovic A., Waagstein F., Gelbrich G., Cvijanovic D., Dungen H.D. (2015). Improvement of Ventricular-Arterial Coupling in Elderly Patients with Heart Failure After Beta Blocker Therapy: Results from the CIBIS-ELD Trial. Cardiovasc. Drugs Ther..

[36] Kolwicz S.C., Purohit S., Tian R. (2013). Cardiac Metabolism and its Interactions with Contraction, Growth, and Survival of Cardiomyocytes. Circ. Res..

[37] Toblli J.E., Cao G., Rivas C., Giani J.F., Dominici F.P. (2016). Intravenous iron sucrose reverses anemia-induced cardiac remodeling, prevents myocardial fibrosis, and improves cardiac function by attenuating oxidative/nitrosative stress and inflammation. Int. J. Cardiol..

