# Supplementary material for: Myocardial Performance Improvement After Iron Replacement in Heart Failure Patients: The IRON-PATH II Echo-Substudy
Source: J Clin Med. 2025 Jun 7;14(12):4048. doi: 10.3390/jcm14124048 (PMC12193777; doi:10.3390/jcm14124048)

Table S1. LV end-systolic elastance (Ees), arterial elastance (Ea) and ventricular–arterial coupling formulas and normal values.

**LV end-systolic elastance (Ees)**
Ees = (DBP – [End (est) × SBP × 0.9)]⁄End (est) × SV
Normal value: 2.3 ± 1.0 mmHg⁄mL
**Arterial elastance (Ea)**
Ea = (SBP × 0.9)⁄SV
Normal value : 2.2 ± 0.8 mmHg⁄mL
**Ventricular–arterial coupling**
Ea/Ees
Normal value 1.0±0.36

DBP: diastolic blood pressure. SBP: systolic blood pressure. SV: stroke volume.

Table S2. Demographic and clinical characteristics of all patients included in this analysis, overall and according to iron status.

	**Whole Cohort (n=222)**	**Echo substudy Cohort (n=98)**	**Non-echo substudy Cohort (n=124)**	**P-value**
Age, years	69 (13)	72 (10)	68 (14)	**0.027**
Sex (female), n (%)	59 (27 %)	22 (22 %)	38 (30 %)	0.229
Systolic blood pressure, mmHg	120 (21)	119 (19)	121 (22)	0.358
Heart rate, bpm	69 (11)	69 (12)	68 (12)	0.558
NYHA functional class, n (%)				0.483
I	49 (22 %)	16 (16%)	32 (25 %)	
II	146 (67 %)	70 (71%)	81 (56 %)	
III	24 (11 %)	10 (10%)	14 (11 %)	
IV	2 (1 %)	2 (2%)	0 (0 %)	
BMI, Kg/m^2	28 (11)	27 (4)	29 (13)	0.146
Ischaemic aetiology of HF, n (%)	103 (47 %)	49 (50%)	54 (44 %)	0.268
Hypertension, n (%)	155 (70 %)	76 (78%)	79 (64 %)	0.034
Diabetes mellitus, n (%)	105 (47 %)	47 (48%)	58 (47 %)	0.798

Previous MI, n (%)	105 (47 %)	50 (51%)	55 (44 %)	0.353
PAD, n (%)	36 (16 %)	15 (15%)	21 (17 %)	0.660
Atrial fibrillation, n (%)	98 (44 %)	49 (50%)	49 (40 %)	0.031
Haemoglobin, g/dL	14.5 (8.4)	14.1 (1.4)	15.1 (1.3)	0.390
Creatinine, umol/L	116.9 (46.8)	124.7 (52.7)	110.8 (40.3)	**0.030**
Estimated glomerular filtration rate, ml/min/1.73m^2{Citation}	58 (21)	55 (21)	60 (21)	**0.044**
Sodium, mmol/L	141 (3)	141 (2)	140 (3)	0.094
Potassium, mmol/L	4.74 (0.45)	4.75 (0.43)	4.74 (0.48)	0.903
Ferritin, ng/mL	184 (173)	244 (204)	138 (127)	**<0.001**
TSAT, %	22 (10)	22 (9)	21 (10)	0.351
Transferrin, umol/L	32.5 (21.1)	32.8 (30.2)	32 (8)	0.698
TIBC, umol/L	62 (15.5)	58 (9.7)	65 (18.4)	**0.001**
NT-proBNP, pg/mL (median, IQR)	2551(1229 – 4122)	1679 (851 – 2889)	2893 (1280-4634)	**0.019**
ARNI, n (%)	174 (78 %)	74 (76%)	100 (81 %)	0.515
ACEI or ARBs, n (%)	25 (11 %)	12 (12%)	13 (10 %)	0.776
Beta-blockers, n (%)	207 (93 %)	92 (94%)	115 (93 %)	0.357
MRA, n (%)	176 (79 %)	74 (76%)	102 (82 %)	0.265
iSGLT2, n (%)	180 (81 %)	70 (71%)	110 (89 %)	0.016
Diuretics, n (%)	152 (68 %)	73 (75%)	79 (64 %)	0.053
Antiplatelet therapy, n (%)	89 (40 %)	38 (39%)	51 (41 %)	0.878
Anticoagulant therapy, n (%)	126 (57 %)	57 (57%)	69 (56 %)	0.598
Cardiac resynchronisation therapy, n (%)	28 (13 %)	9 (9%)	19 (15 %)	0.257
Implantable cardioverter defibrillator device, n (%)	48 (22 %)	15 (15%)	33 (27 %)	0.055

ID: iron deficiency. NYHA: New York heart Association. BMI: body mass index. CV: cardiovascular. HF: heart failure. MI: myocardial infarction. PAD: peripheral arterial disease. ARNI: angiotensin receptor neprilysin inhibitor. ACEi: angiotensin-converting enzyme inhibitor. ARBs: angiotensin receptor

blockers. MRA: mineral corticoid receptor antagonist. MRA: aldosterone receptor antagonist. SGLT2i: sodium–glucose cotransporter 2 inhibitor. TSAT: transferrin saturation. TIBC: total iron binding capacity. IQR: interquartile range.

Figure S1. Flowchart.

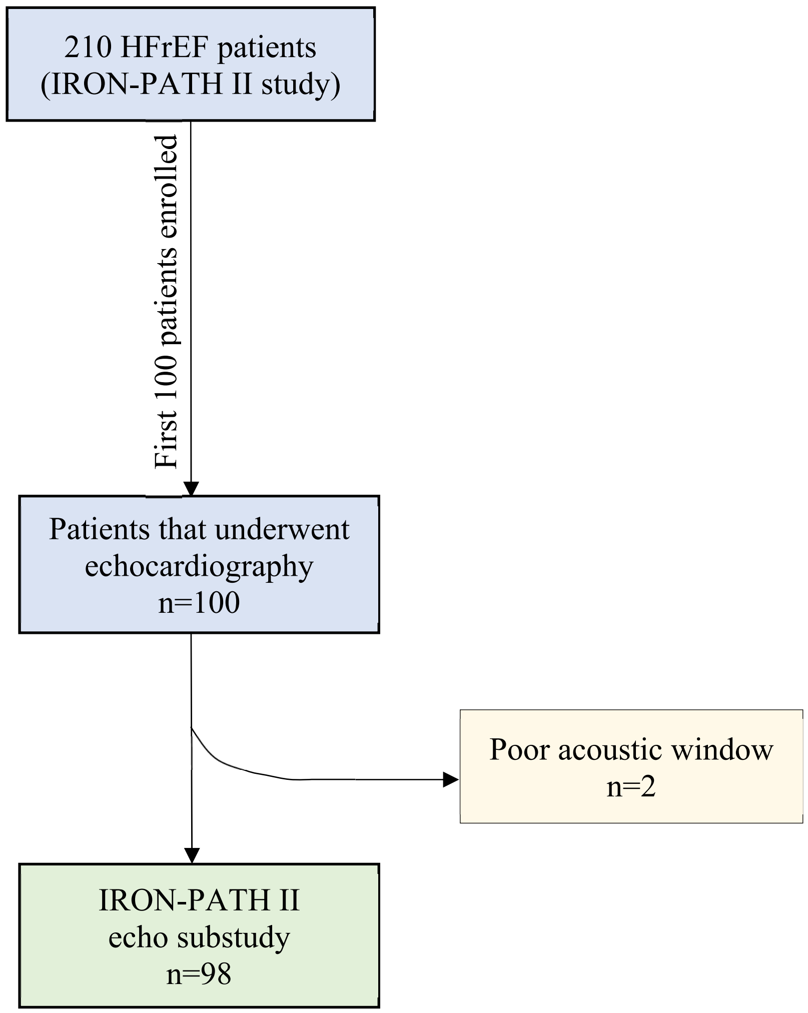

Figure S2. Boxplots (showing mean and standard deviation) of LV myocardial performance (LVEF, GLS, myocardial work, constructive work, wasted work and work efficiency), RV myocardial performance (TAPSE, RV coupling, FAC, RV free wall strain) and ventricular–arterial coupling (systemic arterial resistance, LV elastance, arterial elastance and VAC) according to iron status.

Panel A. Left ventricle performance parameters.

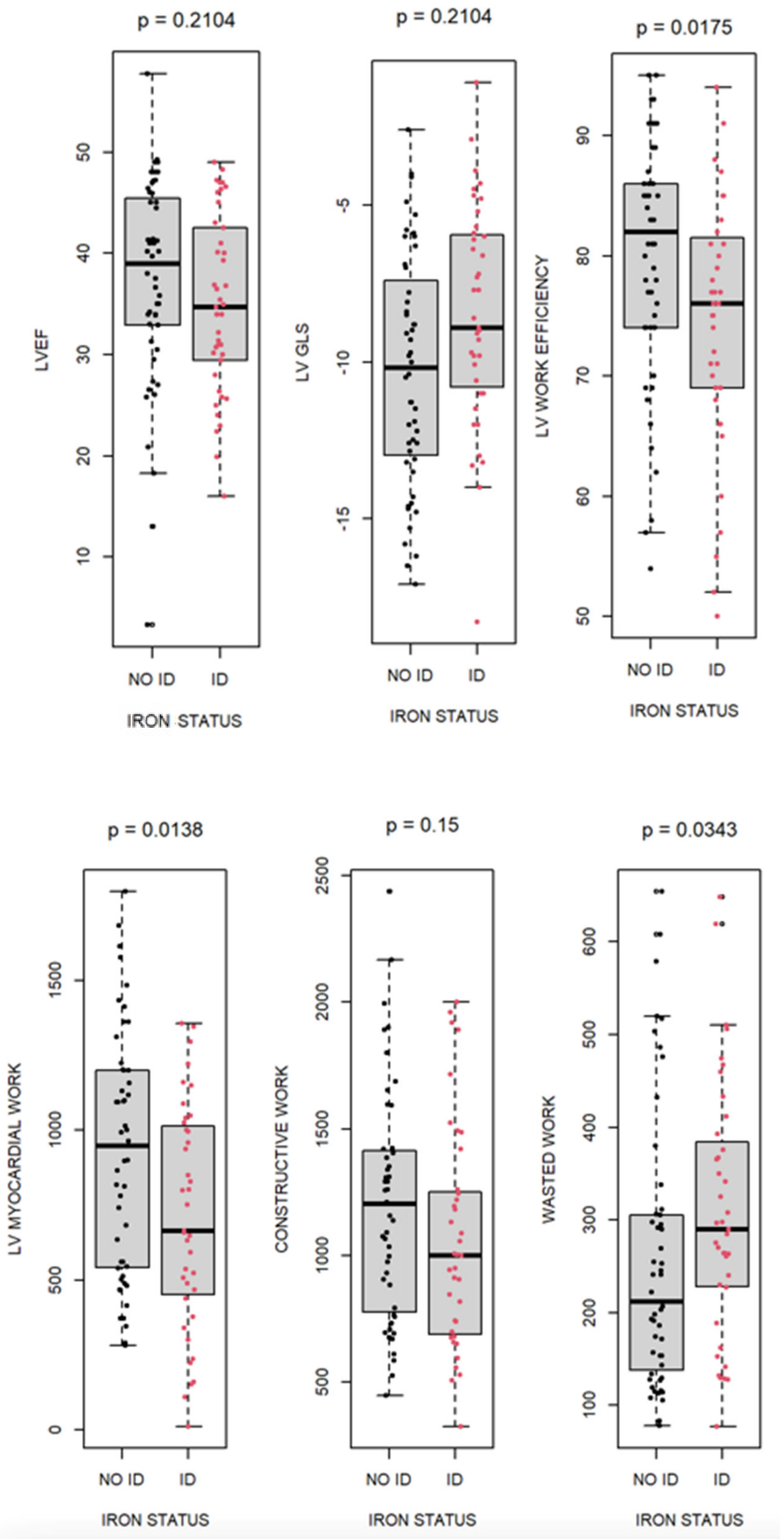

Panel B. Right ventricle performance parameters.

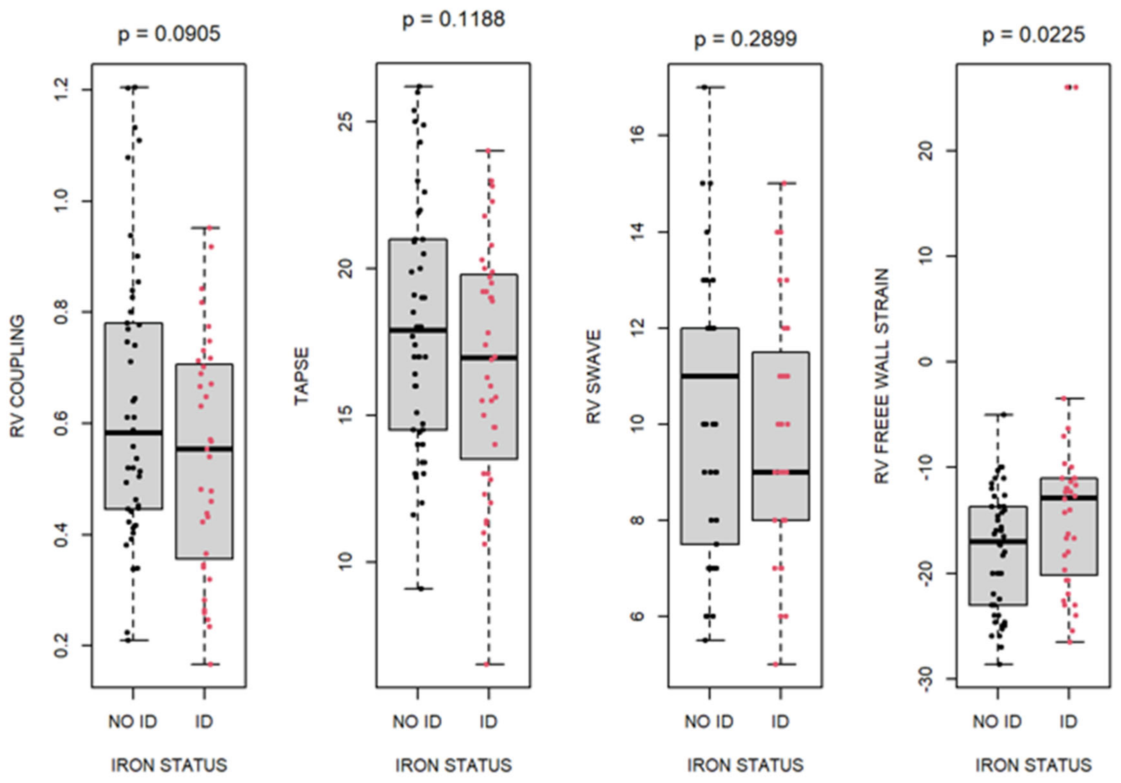

Panel C. Ventricular–arterial coupling parameters.

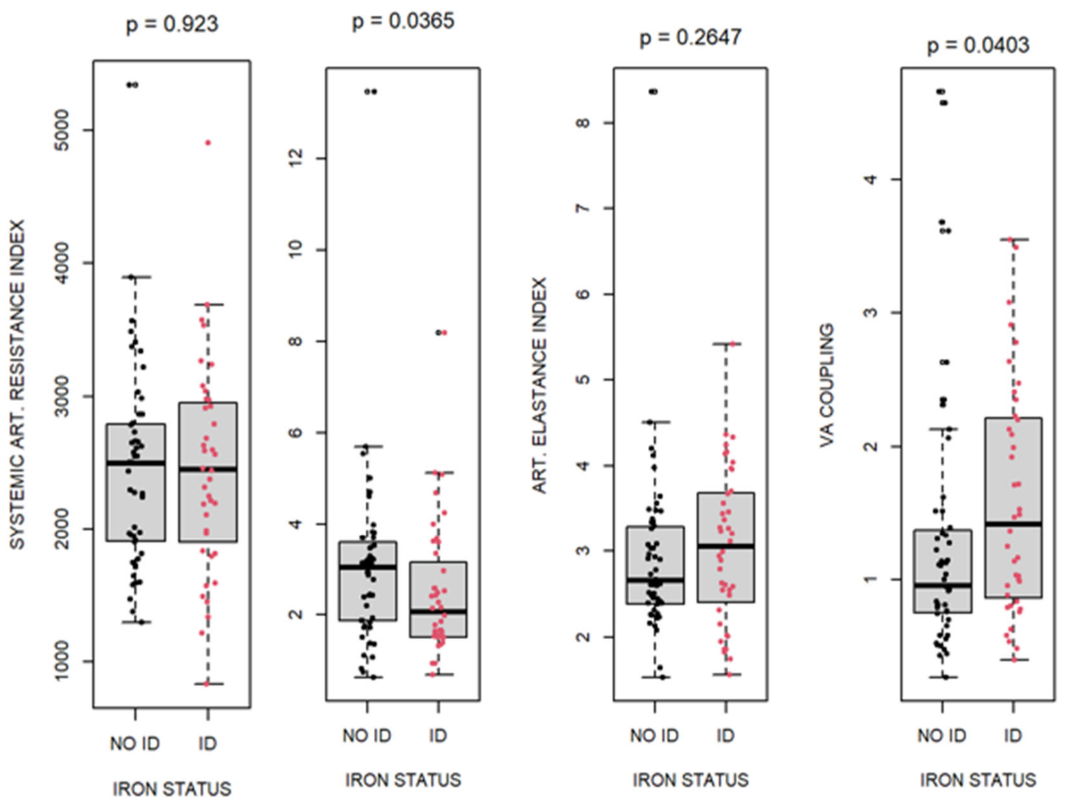

Supplement: Supplementary file 1 [file jcm-14-04048-s001.zip › jcm-3677748-supplementary.pdf]
